# Investigation on the Occurrence of *Aedes* Species in Borderline of Iran and Azerbaijan for Control of Arboviral Diseases

**Published:** 2019-06-24

**Authors:** Eslam Moradi-Asl, Hassan Vatandoost, Davod Adham, Daryosh Emdadi, Hassan Moosa-Kazemi

**Affiliations:** 1 Department of Public Health, School of Public Health, Ardabil University of Medical Sciences, Ardabil, Iran; 2 Department of Medical Entomology and Vector Control, School of Public Health, Tehran University of Medical Sciences, Tehran, Iran; 3 Department of Environmental Chemical Pollutants and Pesticides, Institute for Environmental Research, Tehran University of Medical Sciences, Tehran, Iran; 4 Center for Disease Control, Ardabil University of Medical Sciences, Ardabil, Iran

**Keywords:** Larval habitat, *Aedes*, Borderline, Iran

## Abstract

**Background:**

To investigate the diversity of the genus *Aedes* present in the natural areas of Ardabil Province, north-west of Iran.

**Methods:**

This cross-sectional study was carried out from Apr to Oct 2016 in North-western of Iran. Thirty-three areas of 10 cities which are border areas were selected randomly. The larvae were collected 2 times in each month during the seasonal activities of mosquitoes and the larvae were identified morphologically according to the appropriate identification keys.

**Results:**

Overall, 694 larvae were collected from four counties, from which only 7.2% were *Aedes* larvae. Three species of *Aedes* were identified which include *Ae. caspius*, *Ae. vexans* and *Ae. flavescens*. *Aedes flavescens* is reported from Ardabil Province for the first time.

**Conclusion:**

*Aedes* species were a high density in borderline of Iran and Azerbaijan. Therefore, the north parts of Ardabil Province are a suitable habitat for *Aedes* species mosquitoes. Care should be taken for vector control in the case of occurrence of any arboviruses transmitted by *Aedes* mosquitoes.

## Introduction

Mosquito-borne arboviruses are health threat in the worldwide. For instance, more than 2.5 billion people live in high-risk areas of dengue fever (DF) in the world and over 100 million people are infected with this disease (1, 2).

Currently, there is no vaccine and no specific treatment for DF (3). Recently, outbreaks of DF have happened in Malaysia, Taiwan, and India (4). Endemic DF always occurred in Southeast Asia (5). However, some outbreaks recently have been occurred in parts of the Middle East, South Asian countries including Pakistan (6, 7).

Some factors affecting the distribution of DF in different parts of the world are as follows: Increasing urban population density, increased travel, and unsystematically urbanization (8–10). DF has been reported in 120 countries (11). The agent of DF is a Flaviviridae family and the main vectors are *Aedes aegypti* and *Ae. albopictus* (12). Thus mosquitoes are an invasive species in world widespread in tropical and temperate regions of the world. The ability to lay eggs and grow in dishes cultural artifacts. In the last two decades and facilitate the movement in the world are impacts factors in distribution of this mosquitoes (13).

*Aedes aegypti* and *Ae. albopictus* are vectors of important diseases such as DF, yellow fever and Chikungunya (14–16). *Aedes aegypti* is a mainly urban vector and is feeding exclusively from human (17). *Aedes albopictus* are mostly found in suburban and rural environments and are feeding of the different species of mammals, including humans, as well as the different species of birds (18).

Currently, vector control is the best method to control of the DF (19). In Iran, studies regarding the biodiversity and distribution of *Aedes* are limited. However, there is no information on *Aedes* mosquito’s diversity components in north-western of Iran. The aim of this study was to investigate the diversity of *Aedes* genus present in the natural areas, as well as the differences on the faunestic composition of *Aedes* species in function of the climatic and ecological features of Ardabil Province borderline.

Various *Aedes* mosquito species are considered as potential vectors of Zika virus including *Ae. africanus*, *Ae. albopictus*, *Ae. polynesiensis*, *Ae. unilineatus*, *Ae. vitiates*, *Ae. apicoargenteus*, *Ae. leuteocephalus*, *Ae. aegypti*, *Ae. vitattus*, *Ae. furcifer*, and *Ae. hensilli*.

## Materials and Methods

### Study area

**Fig. 1.**
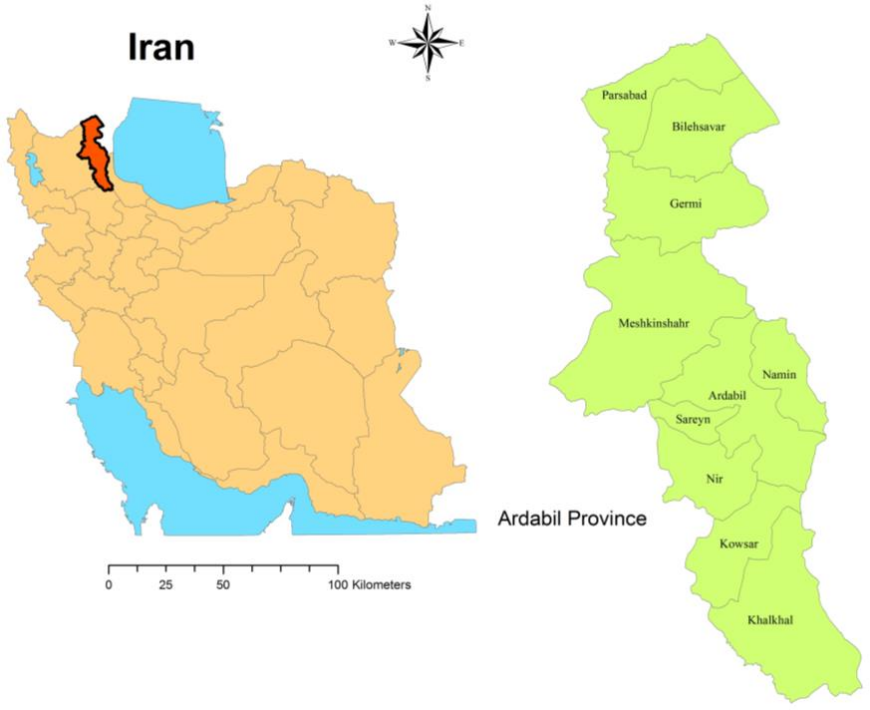
Map of study area, Ardabil Province, Iran

This cross-sectional study was carried out from Apr to Oct 2016 in north-western of Iran. Ardabil Province is located in northwestern Iran 37.45° to 39.42° N and 47.30° to 48.55° E. The province has an area of 17 953km^2^. This province is bordered to the north with the Republic of Azerbaijan and along the border is 282.5km (Fig. 1). In 159km from the border, flowing Aras and Balha rivers. During the border, Iran linked to the Republic of Azerbaijan for two areas Bilehsavar and Aslanduz. Ardabil Province in the longitudinal axis of the expansion (1°35`) and high extent to the north-south latitudes (2°31`) have a large variety of climates. About 2/3 textured mountainous with large variation in height and the rest are flat areas and posts. North province (Mugan plain) with low altitude has relatively warm weather and central and southern regions have a cool mountain climate (20–22).

### Sample collection

Overall, 33 areas of 10 cities of priority border areas were selected randomly. During the seasonal activity, the larvae were collected in each month 2 times. Using a ladle handling, and the standard ladle of 350mL was collected from natural and artificial larval habitats. In each habitat, sampling was collected from different parts and the ladle was made 10 times on each side. In the case of well water used from the bucket and the limited larval habitats such as cavity trees were used from droppers. The larval stages III and were stored in lactophenol solution and after about a week and transparent larvae, using Berlese’s Fluid were prepared microscopic slides and identified morphologically using appropriate identification keys.

## Results

Overall, 2000 mosquito larvae were collected, from which only *Aedes* larvae were selected and identified. From 33 areas, six (18%) were positive for the presence of *Aedes* larvae. In four counties, (40%) *Aedes* larvae were collected. Totally, 694 larvae were collected from four counties that 7.2% were *Aedes* larvae. Three species of *Aedes* genus were identified which included *Ae. caspius*, *Ae vexans*, and *Ae. flavescens*. These species were reported from thee Ardabil Province for the first time. All three species were collected from 78–2114 meter above sea level altitude. *Aedes* larvae were collected from two different climate zones. The first zone: the northern part of the province where the climate is hot and humid and low altitude (60–78km) that includes Pars Abad and Bilehsavar and the second zone: southern part of the province with mountainous climate and high altitude (2114–2110m), which includes the Khalkhal and Sareyn (Table 1).

**Table 1.** Total larvae collected from Ardabil Province, north-western of Iran, 2016

**location**	**Village**	**Total larvae**	***Aedes* larvae**	**Genus**	**Species**	**Elevation**	**Y**	**X**
**Sareyn**	Alvars	40	1	*Aedes*	*caspius*	2110m	38.14985	47.96122
**Bilehsavar**	Jafarabad	68	10	*Aedes*	*caspius*	176m	39.50238	48.04068
			2	*Aedes*	*flavescens*			
**Khalkhal**	khangahe	213	1	*Aedes*	*caspius*	2114m	37.53637	48.5755
			1	*Aedes*	*flavescens*			
**Parsabad**	Oltan	251	3	*Aedes*	*caspius*	74m	39.60545	47.76123
	Mahmoudabad	70	10	*Aedes*	*caspius*	87m	39.54975	47.97872
			3	*Aedes*	*flavescens*			
			3	*Aedes*	*vexans*			
	Normohamadkandi	52	10	*Aedes*	*caspius*	165m	39.4721	47.49537
			5	*Aedes*	*flavescens*			
			1	*Aedes*	*vexans*			
**Total**		694	50					

In the first zone the larvae were collected in Jun and Sep months but in the second zone in Jul and Aug. All three species *Aedes* more in border areas The maximum *Aedes* larvae from three species were collected in borderline of Iran and Azerbaijan but in other areas in this study, only two species for low abundance were collected (Table 2). The survey of larvae habitat characteristics showed that most of larvae were collected (66%) in areas without trees and shrubs and sunny. Totally 80% of larvae habitats were temporary that including holes and marshes around rivers and craters were caused by the overflow waters and 86% were natural habitat. About 50 % of the larvae collected in the afternoon and at cooler temperatures and 33% at mid-day and 17% at AM. The water of larval habitat more was mainly stagnant and 50% transparent and type of bed habitat was 80% clay and 20% sand.

**Table 2.** Larval abundance of *Aedes* in Ardabil Province, north-western of Iran, 2016

**Species**	***Aedes caspius***	***Aedes flavescens***	***Aedes vexans***	**Total**	**%**
**Kowsar**	0	0	0	0	0
**Bilehsavar**	10	2	0	12	24
**Parsabad**	23	8	4	35	70
**Germi**	0	0	0	0	0
**Khalkhal**	1	1	0	2	4
**Nir**	0	0	0	0	0
**Namin**	0	0	0	0	0
**Meshkinshahr**	0	0	0	0	0
**Sareyn**	1	0	0	1	2
**Ardabil**	0	0	0	0	0

## Discussion

In this study three spices of *Aedes* larvae (*Ae. caspius*, *Ae. vexans* and *Ae. flavescens*) identified from Ardabil Province, North-western of Iran that one species (*Ae. flavescens*) was reported for first time. *Aedes* larvae were dispersed in all regions of Ardabil Province but the frequency of *Aedes* larvae were maximum in north regions of this province that borderline of Iran and Azerbaijan. Only two adult species was reported (*Ae. caspius* and *Ae. vexans*) (22). *Aedes caspius* is distributed in Palearctic areas (23) and in Iran reported from more than 16 provinces such as Gilan, West Azerbaijan, Khorasan, Zanjan, Lorestan, Isfahan, Yazd, Kerman, Hormozgan, Bushire and Khozestan (24). This species very more frequently collected from six regions (Parsabad, Aslanduz, Bilehsavar, Khalkhal and Sareyn) in this study that two areas (Aslanduz and Bilehsavar) were located in borderline of Iran and Azerbaijan and both are customhouse. This species is also collected with extensive distribution from Gilan and Ardabil Province (25–26). Moreover, *Ae. caspius* reported from Bushire (27), Eastern of Iran (28), Chaharmahal and Bakhtiari (29), East Azerbaijan (30) and Qom Province (31). In 2016, *Ae. caspius* along with *Ae. albopictus* and *Aedes unilineatus* reported from Sistan and Baluchestan (32). *Aedes caspius* more feeding on mammals and humans (9, 33) and can be transmitted Rift Valley fever, dirofilariasis and tularemia (34).

*Aedes vexans* is distributed in Holoarctic and Oriental areas (35). This species and *Ae. eagypti* and *Culex quinquefasciatus* have the most distribution in world (33). In Iran also reported from Gillan, West Azerbaijan, Mazandaran, Bushire and Hormozgan (23). In this study *Ae. vexans* larvae were collected 2 times in Jun and Sep from Parsabad and Aslanduz. The number of this species were lower in comparison to other *Aedes* species but in Gillan Province the most larvae collected were *Ae. vexans* (25). The results of this study showed that the larvae of *Ae. vexans* were collected of less than 200m altitude that this result matched to another study (22) and the adults of this species collected from east Azerbaijan in high altitude (30). The feeding preference of this species is on the mammals then birds and reptiles (32). *Aedes vexans* can transmitted various diseases such as eastern and western equine encephalitis, Japanese encephalitis and California encephalitis.

*Aedes flavescens* was reported first time in Ardabil Province and 11 larvae of *Ae. Flavescens* collected from three parts of this area (Parsabad, Bilehsavar and Khalkhal). Totally 100% of larvae habitats were temporary and most whit vegetation. Total of these larvae were collected at afternoon. Zaim et al. for first time reported *Ae. flavescens* from West Azerbaijan in 1987 and one time from large pool whit vegetation (24). These results showed that *Ae. flavescens* were dispersed in north-west of Iran.

## Conclusion

*Aedes* species were a high density in borderline of Iran and Azerbaijan. 40% of Ardabil Province was found *Aedes* mosquitoes. So the north parts of Ardabil Province are a suitable habitat for *Aedes* species mosquitoes. Therefore, more studies need to be done in these areas.
